# Seasonal variation in the stomach microbiota of two sympatrically breeding Pygoscelis penguin species at Signy Island, South Orkney Islands

**DOI:** 10.1099/mic.0.001503

**Published:** 2024-09-26

**Authors:** Wen Chyin Yew, Stacey Adlard, Michael James Dunn, Siti Aisyah Alias, David Anthony Pearce, Azizan Abu Samah, Peter Convey

**Affiliations:** 1 Department of Applied Sciences, Faculty of Health and Life Sciences, University of Northumbria, Newcastle upon Tyne, UK; 2 National Antarctic Research Center, University of Malaya, Kuala Lumpur, Malaysia; 3 Institute of Ocean and Earth Sciences, University of Malaya, Kuala Lumpur, Malaysia; 4 British Antarctic Survey, Natural Environmental Research Council, Cambridge, UK; 5 Department of Zoology, University of Johannesburg, Auckland Park, South Africa; 6 Millennium Institute – Biodiversity of Antarctic and Sub-Antarctic Ecosystems (BASE), Santiago, Chile

**Keywords:** Antarctic krill, diet, microbiome, penguins, sea ice, seasonal variation

## Abstract

The gut microbiomes of Antarctic penguins are important for the fitness of the host birds and their chicks. The compositions of microbial communities in Antarctic penguin guts are strongly associated with the birds’ diet, physiological adaptation and phylogeny. Whilst seasonal changes in food resources, distribution and population parameters of Antarctic penguins have been well addressed, little research is available on the stability or variability of penguin stomach microbiomes over time. Here, we focused on two *Pygoscelis* penguin species breeding sympatrically in the maritime Antarctic and analysed their stomach contents to assess whether penguin gut microbiota differed over three austral summer breeding seasons. We used a high-throughput DNA sequencing approach to study bacterial diversity in stomach regurgitates of Adélie (*Pygoscelis adeliae*) and chinstrap (*Pygoscelis antarctica*) penguins that have a similar foraging regime on Signy Island (South Orkney Islands). Our data revealed significant differences in bacterial alpha and beta diversity between the study seasons. We also identified bacterial genera that were significantly associated with specific breeding seasons, diet compositions, chick-rearing stages and sampling events. This study provides a baseline for establishing future monitoring of penguin gut microbiomes in a rapidly changing environment.

**Impact Statement**

This study makes a significant contribution to the field by documenting seasonal variations in the stomach microbiota of two sympatrically breeding Antarctic penguins on Signy Island (South Orkney Islands). Our data underscore the complexity of microbial dynamics in a seemingly homogeneous environment and highlight the influence of seasonal changes on penguin gut microbiomes. Notably, despite similar foraging patterns and environmental conditions, distinct seasonal shifts in bacterial diversity were observed in the stomach regurgitates, suggesting a nuanced interplay between diet, environmental factors and microbial communities. By drawing parallels with previous research on alpine accentors and emphasizing the impact of climate-driven alterations in penguin diet and sea ice duration, this study expands the understanding of ecological drivers shaping stomach microbial community composition. Moreover, the identification of specific bacterial taxa associated with diet preferences and chick-rearing stages unveils potential biomarkers and ecological indicators for monitoring environmental changes. This research provides essential baseline data for future investigations into the adaptive capacity of penguin gut microbiomes amidst ongoing climate change. It underscores the significance of microbial ecology in polar environments and the need for continued monitoring to assess the resilience of Antarctic ecosystems.

## Data Summary

The authors confirm that all supporting data, code and protocols have been provided within the article or through supplementary data files.

## Introduction

In birds (including penguins), the term ‘gut’ refers to portions of the alimentary tract, which begins with the crop/stomach and ends with the anus/cloaca [1]. The gut microbiome and its variability in Antarctic penguins play a significant role in transforming food resources into energy and nutrients required for the growth, maintenance of health and reproductive success of the host birds [2,3]. The gut microbes can also be transferred to chicks via regurgitation during feeding, in turn benefiting their growth [4]. The succession of microbes in the guts of Antarctic penguins is influenced by their diets [5], physiological adaptation to the surrounding environments [6,8] and phylogeny [2,9]. However, these penguin gut microbiomes were achieved from either cloacal or guano samples and may not serve as a good representative for the stomach microbiome [10]. Previously, we used penguin stomach contents to assess their gut microbiota and reported inter-specific variation in the gut microbiota of Adélie (*Pygoscelis adeliae*) and chinstrap (*Pygoscelis antarctica*) penguins that consumed a diet consisting entirely of Antarctic krill (*Euphausia superba*) in overlapping breeding and foraging environments at Signy Island [11]. Here, we investigated if the variability in penguin stomach microbiota persists across different breeding seasons.

Signy Island, as part of the South Orkney Islands archipelago in the maritime Antarctic, has experienced rapid warming of air temperatures since the 1950s [12]. The island hosts breeding populations of around 18 000 pairs of both Adélie and chinstrap penguins [13,14]. A long-term monitoring programme of Antarctic penguins at Signy Island has reported a significant decline in the population sizes of Adélie and chinstrap penguins over the past six decades, potentially influenced by several large-scale processes driven by changes initiated through global climate forcing, including variation in sea ice conditions, prey recruitment and over-winter juvenile survival [13,16]. Ongoing climatic changes in this region have caused reductions in thickness and the extent of seasonal sea ice [17,18], consequently affecting the life cycle and winter survival of Antarctic krill, which are highly dependent on the sea ice habitat [19,20]. Since the main diet of Adélie and chinstrap penguin breeding at Signy Island is Antarctic krill, changes in their access to this resource have led to alterations in their diets [21,22] and physiological characteristics [23,25], affecting their reproductive performance [26]. In this study, we set out to characterize and compare bacterial community composition in the stomach regurgitates obtained from Adélie and chinstrap penguins on Signy Island across three austral summer breeding seasons, using a high-throughput DNA sequencing approach.

## Methods

### Study site and sample collection

This study was conducted during the guard and crèche chick-rearing period of the two penguin species in the breeding seasons 2011/2012, 2013/2014 and 2014/2015 on the Gourlay Peninsula (60°43.5860′S, 45°35.0630′W), Signy Island, South Orkney Islands (Fig. S1, available in the online Supplementary Material). As part of the long-term monitoring programme of the Convention for the Conservation of Antarctic Marine Living Resources (CCAMLR) Ecosystem Monitoring Programme (CEMP), five or six healthy adult *Pygoscelis* penguins that had just returned from the sea were captured on up to ten sampling events each breeding season to determine their diet composition following CEMP Standard Method A8 [27,29]. The captured birds were marked with non-hazardous spray ink to ensure that no birds were sampled more than once within a breeding season. The procedures used are approved by both the British Antarctic Survey (BAS) and CCAMLR ethical committees. Penguin stomach regurgitates were aseptically obtained from the stomach and collected into sterile 50-ml Falcon tubes, as previously described by Yew *et al*. [11]. Samples were then stored and transported at −20 °C to BAS under the UK Department for Environment, Food and Rural Affairs import licence for further DNA extraction. Penguin diet composition was calculated as the relative abundance from the CCAMLR-CEMP quantitative analysis on prey components in the stomach regurgitates of each captured bird [28,29].

### DNA extraction, V4-16S gene amplification and sequencing

All laboratory work was conducted in an Astec-Microflow class II Biosafety cabinet (Bioquell UK Ltd., UK) using nuclease-free or sterilized consumables and equipment. Genomic DNA of a total of 54 randomly selected penguin stomach regurgitate samples was extracted using the DNeasy Blood and Tissue Kit following the manufacturer’s protocol (QIAGEN, Germany). The V4-16S rRNA gene fragments were amplified with adapted forward and reverse primers 515F and 806R under the PCR conditions described by Caporaso *et al.* [30]. A kit negative control was included in every DNA extraction and V4-16S rRNA gene amplification. The quality and the quantity of purified amplicons were examined using a NanoDrop 2000c Spectrophotometer (Thermo Scientific, USA) and Qubit 2.0 Fluorometer (Invitrogen, USA), respectively. In NU-OMICS (Northumbria University, UK), DNA libraries were generated using the Nextera XT DNA Library Preparation Kit (Illumina, USA) and sequenced using Illumina MiSeq version 2500-cycle pair-end run.

### Data processing and bioinformatic analyses

The generated raw sequence data were demultiplexed and subjected to Illumina adapter removal using MiSeq Reporter Software version 2.5 (Illumina, USA). The quality of the paired-end reads was examined using FastQC version 0.11.5 [31], merged and trimmed at Phred Score Q30 using Trimmomatic [32]. Chimeric sequences were identified and removed using USEARCH version 6.1 default parameters [33]. Operational taxonomic unit (OTU) picking was performed using the QIIME two-step open-reference method [34] and assigned using the Greengenes database [35] with a similarity threshold of 97% [36].

To reduce sample heterogeneity for microbiota comparisons, low-count OTUs were further filtered using the default parameter (minimum count, 4; sample prevalence, 20%) and normalized to the minimum library size of samples prior to statistical analyses and visualization in MicrobiomeAnalyst [37]. Rarefaction curves were constructed to examine sampling coverage. We considered bacterial genera with a relative abundance of ≥1% as the dominant community and those bacterial genera that were present in ≥50% of the studied samples as the core community.

Microbiota comparisons were performed based on bacterial alpha (i.e. Shannon index) and beta (Bray–Curtis distance matrix) diversity at the genus classification level rather than the species or OTU level [38]. Non-parametric pairwise tests were used to statistically analyse both alpha and beta diversity values. Principle coordinate analysis was carried out to visualize the bacterial divergence patterns between the compared groups. To identify specific bacterial genera that were significantly associated with a comparison group, biomarker discovery with linear discriminant analysis effect size (LEfSe) was used, with a *P*-value threshold of 0.05 and linear discriminant analysis (LDA) score of 2.0.

## Results

### The core microbiota in *Pygoscelis* penguin stomach regurgitates across breeding seasons

Between 13  375 and 239 954 sequence reads were obtained per sample (Table S1). After data filtering and normalization, the rarefaction curves of all 54 samples approached saturation (Fig. S2), indicating sufficient sample coverage to undertake bacterial community composition analysis. The results were further supported by the calculation of Good’s coverage [39], showing that OTU sampling completeness for all samples was >99% (Table S1). The OTU data were assigned to a total of 10 bacterial phyla and 37 genera.

**Fig. 1.**
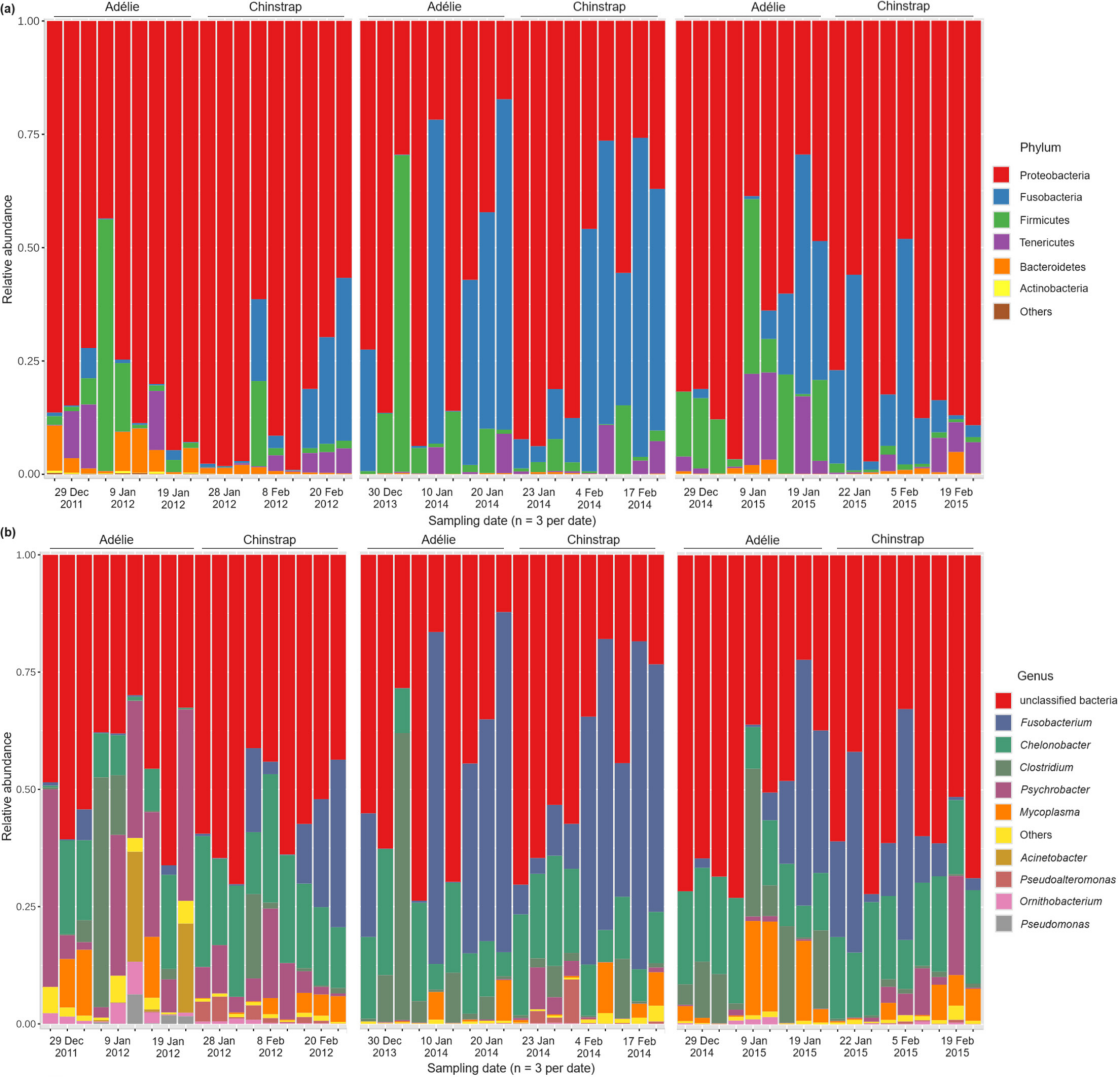
Relative abundances of the frequently encountered bacterial (**a**) phyla and (**b**) genera in the stomach regurgitates of Adélie and chinstrap penguins collected at each sampling event across the three penguin breeding seasons.

The dominant and core bacterial phyla present in the stomach regurgitates of both penguin species were *Proteobacteria* (71%), followed by *Fusobacteria* (17%), *Firmicutes* (7%), *Tenericutes* (3%) and *Bacteroidetes* (1%) (Fig. 1a). At the genus level, *Fusobacterium* (17%), *Chelonobacter* (15%), *Clostridium* (6%), *Psychrobacter* (6%) and *Mycoplasma* (3%) were present in >50% of the study samples (Fig. 1b). Nevertheless, a distinct seasonal shift in the composition of dominant and core bacterial communities was observed in the study samples (Fig. 1).

### Inter-species comparison of penguin stomach microbiota

**Fig. 2.**
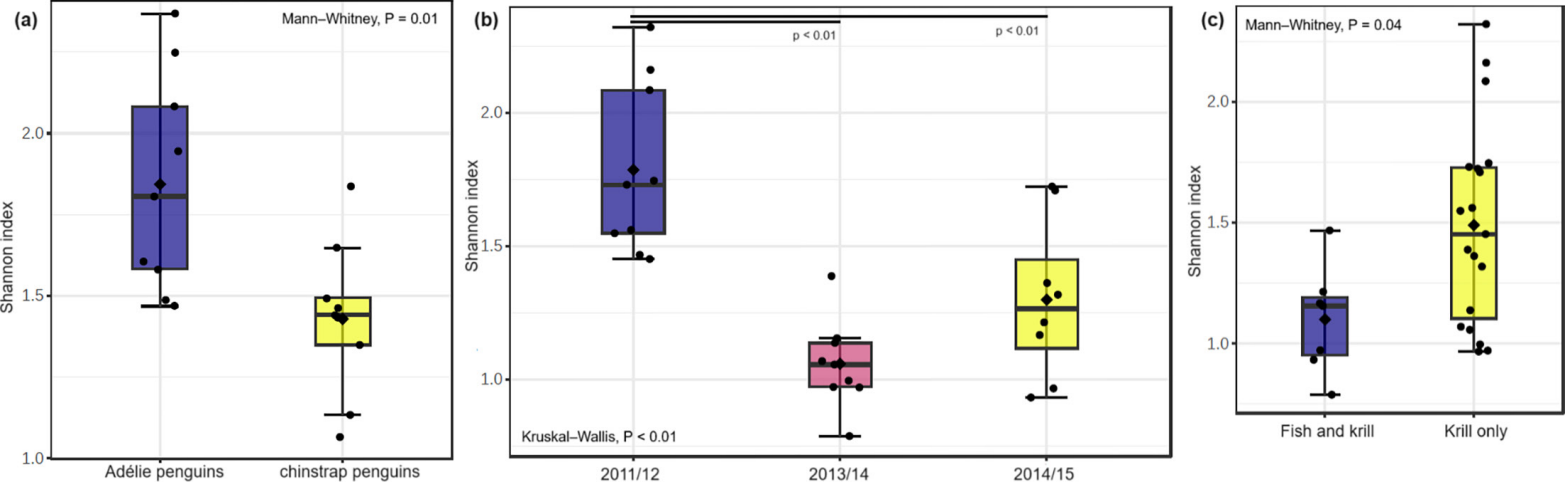
Comparison of alpha diversity values between (**a**) Adélie and chinstrap penguins, (**b**) different breeding seasons of Adélie penguins and (**c**) different diet compositions of Adélie penguins.

We found no significant differences in the bacterial alpha and beta diversity between Adélie and chinstrap penguin stomach regurgitates (Table S2). Of the three study seasons, bacterial communities in Adélie penguin stomach regurgitates collected in the 2011/2012 season were significantly more diverse than those in chinstrap penguins (Figs2a  3a).

**Fig. 3.**
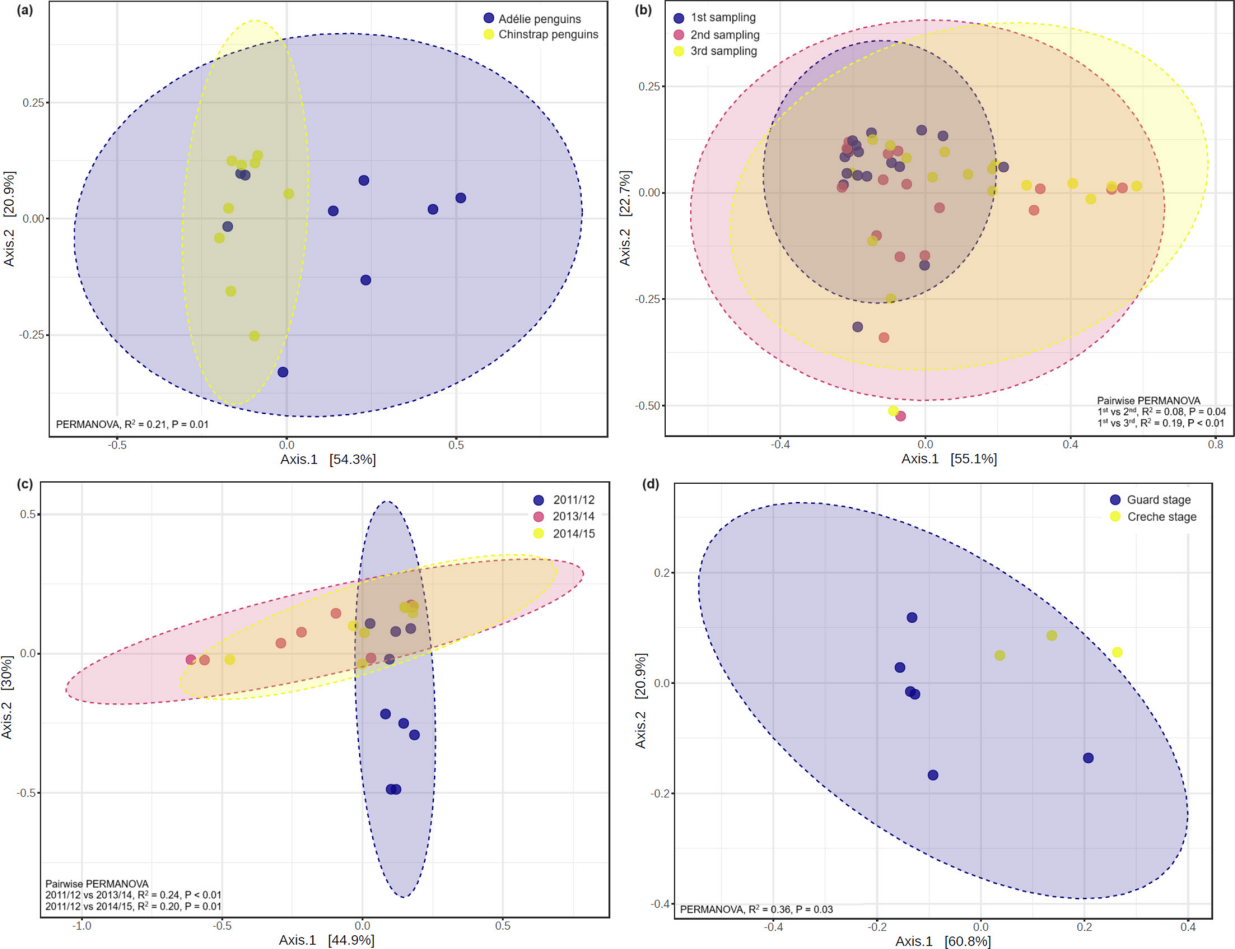
Comparison of beta diversity values between (**a**) Adélie and chinstrap penguins, (**b**) different sampling events of both penguin species, (**c**) different breeding seasons of Adélie penguins and (**d**) different chick-rearing stages of chinstrap penguins in the breeding season 2011/2012.

### Intra-seasonal variation in penguin stomach microbiota

**Fig. 4.**
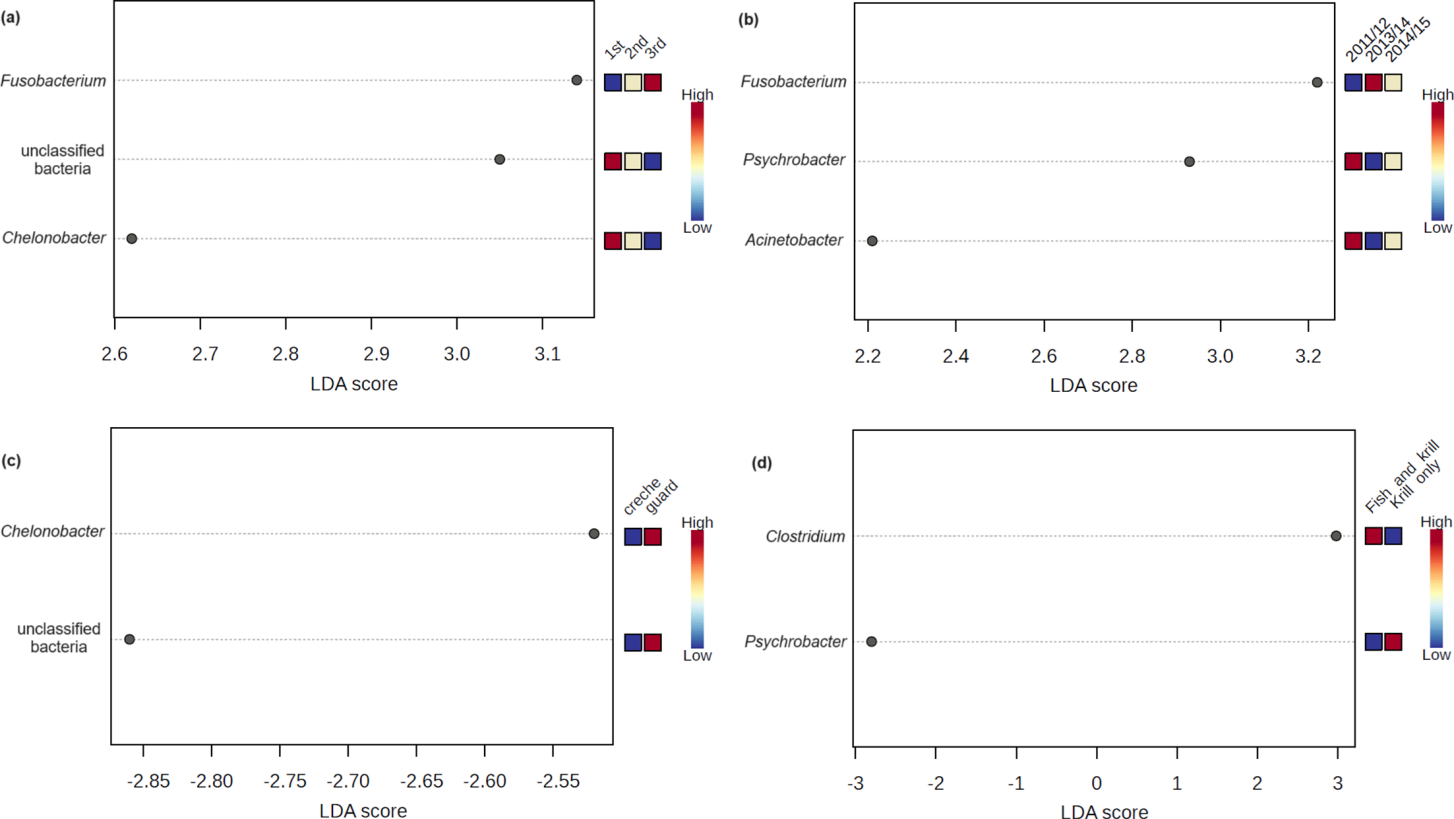
LEfSe shows bacterial genera that were significantly associated with the different (**a**) sampling events, (**b**) breeding seasons, (**c**) chick-rearing stages and (**d**) diet compositions. The genera are ranked in increasing order of LDA scores as shown on the *X*-axis, whilst the heatmap at the right of the plot indicates whether the abundance of a genus was higher (towards red) or lower (towards blue).

Overall, both *Pygoscelis* penguins showed no significant differences in bacterial alpha diversity values between the three sampling events within or across the study seasons (Table S2). The 2013/2014 season had a wider range of alpha diversity values (difference in Shannon index = 1.56) than those in 2011/2012 (0.93) and 2014/2015 (1.16). However, pairwise permutational analysis of variance (PERMANOVA) showed that bacterial community compositions at the first sampling event differed significantly from those collected at the subsequent sampling events (Fig. 3b). In addition, a significantly greater relative abundance of *Chelonobacter* was observed in the first sampling event for both penguin species than the other sampling events, whilst *Fusobacterium* showed a significantly greater abundance in the third sampling event (Fig. 4a).

### Inter-seasonal variation in penguin stomach microbiota

Adélie (but not chinstrap) penguins showed significant differences in both bacterial alpha and beta diversity values between the three study seasons (Table S2). The most diverse bacterial communities were observed in samples collected during the 2011/2012 season (average Shannon index = 1.80), followed by 2014/2015 (1.32) and 2013/2014 (1.07) (Fig. 2b). Similarly, the compositions of Adélie penguin stomach bacterial communities during the 2011/2012 breeding season differed significantly from those in the subsequent seasons (Fig. 3c). In addition, we found significantly greater relative abundances of *Psychrobacter* and *Acinetobacter* in 2011/2012 than the other two study seasons, whilst a significantly greater relative abundance of *Fusobacterium* was observed in 2013/2014 (Fig. 4b).

### Penguin stomach microbiota and association with their chick-rearing stages

We were able to sample Adélie penguins during the guard (*n*=6) and crèche (*n*=12) stages of the chick-rearing period in the 2011/2012 and 2014/2015 seasons but only those in the crèche (*n*=9) stage in the 2013/2014 season (Table S1). Omitting samples collected in the 2013/2014 season, no significant differences were found in the alpha and beta diversity values between the guard and crèche stages of the chick-rearing period (Table S2). In chinstrap penguins, both the guard (*n*=18) and crèche (*n*=9) stages were sampled in all three seasons. No significant differences in bacterial alpha diversity values were observed between the chick-rearing stages in all three study seasons (Table S2). However, beta diversity was significantly different between the chick-rearing stages in the 2011/2012 season but not in the subsequent seasons (Fig. 3d). Overall, we found a significantly greater relative abundance of *Chelonobacter* in the guard chick-rearing stage compared with the crèche stage (Fig. 4c).

### Penguin stomach microbiota and association with diet composition

The percentage (by mass) of Antarctic krill contained in the stomach regurgitates of each penguin sampled is listed in Table S1. The highest proportion of individuals that consumed 100% krill was found in the 2011/2012 breeding season (94%), followed by 2014/2015 (83%) and 2013/2014 (78%). These data are consistent with the CCAMLR-CEMP complete diet analysis of a total of 182 penguins captured in the same breeding seasons [28,29], with the proportions of penguins consuming 100% krill being 92%, 90% and 77%, respectively. Of the 27 Adélie penguin stomach regurgitates collected across the three study seasons, 20 (74%) consumed 100% krill in their diet, whilst 7 birds (26%) consumed between 1% and 99% krill in their diet, with the remainder being fish. Of the 27 studied chinstrap penguins, 26 (96%) consumed 100% Antarctic krill, and only 1 bird (4%) showed a more varied diet composition. Therefore, we focused on the stomach bacterial community compositions of Adélie penguins and associations with their diet compositions. Adélie penguins that consumed 100% krill showed significantly higher alpha diversity values than those with a more varied diet composition (Fig. 2c). However, no significant difference was found in the beta diversity values between penguins having a different diet composition (Table S2). The genus *Psychrobacter* was significantly more abundant in birds that consumed 100% krill, whilst *Clostridium* was significantly more abundant in birds with a more varied diet composition (Fig. 4d).

## Discussion

We provide the first report of seasonal variation in the stomach microbiota of Antarctic penguins. Across three study seasons, we confirmed significant inter-seasonal variations in both alpha and beta diversity in the stomach bacterial community compositions of Adélie (but not chinstrap) penguins. In an analogous study, Janiga *et al.* [40] reported inter-seasonal variation in the gut microbiota of alpine accentors (*Prunella collaris*) in the Western Carpathian Mountains of Slovakia. They documented that the prevalence of certain bacteria varied between seasons, suggesting that this could have been caused by changes in the diet preferences of the study birds and changes in temperature from spring to winter. In Antarctica, inter-annual variation has previously been reported in penguin diet composition due to the responses of penguins to changes in regional climate and the availability of krill [21,22, 41]. Although inconsistent handling personnel or methodology application between sampling seasons could possibly induce seasonal variation in the stomach microbiota, all samples in this study were collected and processed by the same well-trained personnel following CEMP Standard Method A8 with aseptic techniques. Therefore, we believe that the variation in stomach microbiota reported here is unlikely to be due to inconsistency in sample handling and processing.

In the current study, we found that the highest proportion of *Pygoscelis* penguins consumed only krill in the 2011/2012 breeding season (94%), followed by 2014/2015 (83%) and 2013/2014 (78%). Similarly, the highest diversity of penguin stomach bacterial communities was detected in the 2011/2012 season (mean±se=1.58±0.07), followed by 2014/2015 (1.28±0.07) and 2013/2014 (1.14±0.05). Furthermore, we found that bacterial diversity in the stomach regurgitates of Adélie penguins that consumed only krill was significantly greater than in those with a more varied diet. The South Orkney Fast-Ice series long-term monitoring of sea ice concentration [42] indicates that sea ice was present around Signy Island between the austral autumn of the year preceding each breeding season until the complete fast-ice breakout in the austral spring of the actual breeding seasons of 2011/2012, 2014/2015 and 2013/2014 for durations of 207 days, 155 days and 115 days, respectively. The inter-seasonal variation observed in the penguin stomach bacterial community compositions may be associated with the resource availability of Antarctic krill and the seasonal changes in sea ice duration and concentration. However, further research is required to confirm these potential links.

Although bacterial gene abundance produced by high-throughput sequencing is only semi-quantitative as the approach depends on both the frequency of sequences and cycle of primer binding, we identified a significant association between bacterial assemblages and the sampling events, breeding seasons, diet compositions and chick-rearing stages. The genera *Psychrobacter* and *Acinetobacter* dominated in penguin stomach regurgitates collected in the 2011/2012 season but not in the other two seasons. We also noted a significantly greater proportion of the genus *Psychrobacter* in regurgitates from penguins that consumed only krill. As the stomach regurgitates in this study were obtained from penguins that had just returned from feeding in the sea, these bacteria may also be from the undigested food itself, i.e. the gut and exoskeleton microbiome of the krill. This finding is consistent with previous reports of the abundance of *Psychrobacter* and *Acinetobacter* in Antarctic krill samples and suggests that these bacteria may play a role in releasing enzymes for krill degradation [43,44].

Conversely, we found a significantly greater proportion of *Chelonobacter* in the first sampling event and in the guard chick-rearing stage. *Chelonobacter* was first described from diseased tortoises [45] but has not previously been reported in penguin or other avian gut samples. As penguins in the guard chick-rearing stage were generally captured in the first sampling event across the breeding seasons, the significant differences in the abundance of *Chelonobacter* could be further studied as a potential biomarker for the specific environment.

In addition to environment-specific bacteria, we also identified dominant and core bacterial genera in both penguin species across all three study seasons. These included members of *Fusobacterium*, *Clostridium*, *Psychrobacter* and *Mycoplasma*. The stability of these bacterial communities in the face of temporal variability in the diet composition, physiological characteristics and host phylogeny of the penguins may play a crucial role. These bacteria have previously been reported as common inhabitants in the guts of penguins and may play roles in food digestion and the release of nutrients to the host birds [7,9, 46]. The improved health and growth of the host birds would consequently promote their reproductive success [47] and the health of their chicks. In penguin chicks, the succession of gut microbes begins during egg incubation [48], and it is highly influenced by their diet [5]. The stomach microbes of adult penguins can be transferred to their chicks via regurgitation during feeding, in turn benefiting their chick growth and health [4].

In summary, this study revealed significant seasonal variation in the stomach bacterial community composition of *Pygoscelis* penguins with shared temporal and spatial breeding and foraging ranges on Signy Island. Our findings provide a baseline for the future monitoring of penguin gut microbiomes in a rapidly changing environment.

## supplementary material

10.1099/mic.0.001503 Uncited Supplementary Material 1.

## Abbreviations

BAS: British Antarctic Survey
CCAMLR: convention for the conservation of antarctic marine living resources
CEMP: CCAMLR Ecosystem Monitoring Programme
LDA: linear discriminant analysis
LEfSe: linear discriminant analysis effect size
OTU: operational taxonomic unit
PERMANOVA: permutational analysis of variance
