# Supplementary material for: Seasonal variation in the stomach microbiota of two sympatrically breeding Pygoscelis penguin species at Signy Island, South Orkney Islands
Source: Microbiology (Reading). 2024 Sep 26;170(9):001503. doi: 10.1099/mic.0.001503 (PMC11541225; doi:10.1099/mic.0.001503)
Supplement: Uncited Supplementary Material 1. [file mic-170-01503-s001.pdf]

Table S1. Metadata of the collected samples in this study.

Sample number	Accession number	Sampling date	Penguin species	Chick rearing stage	Krill (%)	Fish (%)	Sampling time point	Breeding season	Library size (counts)	Good's coverage	Shannon index
1	4705294.3	29/12/2011	Adélie	guard	100	0	2012_1st	2011/12	55,322	99.98	1.74
2	4604111.3	29/12/2011	Adélie	guard	100	0	2012_1st	2011/12	87,542	99.96	1.56
3	4604112.3	29/12/2011	Adélie	guard	100	0	2012_1st	2011/12	89,364	99.92	1.57
4	4606031.3	09/01/2012	Adélie	creche	96	4	2012_2nd	2011/12	88,711	99.99	1.48
5	4606032.3	09/01/2012	Adélie	creche	100	0	2012_2nd	2011/12	42,248	99.99	2.36
6	4604221.3	09/01/2012	Adélie	creche	100	0	2012_2nd	2011/12	17,125	99.99	2.07
7	4604222.3	19/01/2012	Adélie	creche	100	0	2012_3rd	2011/12	27,058	99.99	2.13
8	4606033.3	19/01/2012	Adélie	creche	100	0	2012_3rd	2011/12	28,658	99.96	1.55
9	4604104.3	19/01/2012	Adélie	creche	100	0	2012_3rd	2011/12	45,575	99.99	1.77
10	4705469.3	28/01/2012	Chinstrap	guard	100	0	2012_1st	2011/12	62,067	99.95	1.43
11	4705499.3	28/01/2012	Chinstrap	guard	100	0	2012_1st	2011/12	65,716	99.91	1.56
12	4705445.3	28/01/2012	Chinstrap	guard	100	0	2012_1st	2011/12	85,486	99.91	1.05
13	4705630.3	08/02/2012	Chinstrap	guard	100	0	2012_2nd	2011/12	79,833	99.92	1.81
14	4705611.3	08/02/2012	Chinstrap	guard	100	0	2012_2nd	2011/12	51,572	99.91	1.77
15	4705444.3	08/02/2012	Chinstrap	guard	100	0	2012_2nd	2011/12	59,816	99.93	1.24
16	4705461.3	20/02/2012	Chinstrap	creche	100	0	2012_3rd	2011/12	63,729	99.93	1.51
17	4705614.3	20/02/2012	Chinstrap	creche	100	0	2012_3rd	2011/12	41,649	99.93	1.49
18	4705603.3	20/02/2012	Chinstrap	creche	100	0	2012_3rd	2011/12	44,144	99.94	1.36
19	4705524.3	30/12/2013	Adélie	creche	100	0	2014_1st	2013/14	37,485	99.97	1.06
20	4709469.3	30/12/2013	Adélie	creche	100	0	2014_1st	2013/14	112,902	99.96	1.08
21	4715575.3	30/12/2013	Adélie	creche	1	4	2014_1st	2013/14	80,730	99.93	1.18
22	4705625.3	10/01/2014	Adélie	creche	99	1	2014_2nd	2013/14	56,063	99.96	0.80
23	4705525.3	10/01/2014	Adélie	creche	71	20	2014_2nd	2013/14	34,551	99.99	1.01
24	4705597.3	10/01/2014	Adélie	creche	100	0	2014_2nd	2013/14	40,543	99.95	0.97
25	4715573.3	20/01/2014	Adélie	creche	100	0	2014_3rd	2013/14	42,752	99.96	1.16
26	4715572.3	20/01/2014	Adélie	creche	100	0	2014_3rd	2013/14	76,007	99.93	1.40
27	4705483.3	20/01/2014	Adélie	creche	100	0	2014_3rd	2013/14	101,918	99.95	1.01
28	4715576.3	23/01/2014	Chinstrap	guard	100	0	2014_1st	2013/14	45,521	99.98	0.86
29	4705526.3	23/01/2014	Chinstrap	guard	100	0	2014_1st	2013/14	56,854	99.94	1.41
30	4705452.3	23/01/2014	Chinstrap	guard	100	0	2014_1st	2013/14	239,954	99.94	1.41
31	4705618.3	04/02/2014	Chinstrap	guard	100	0	2014_2nd	2013/14	56,039	99.94	1.48
32	4705575.3	04/02/2014	Chinstrap	guard	100	0	2014_2nd	2013/14	59,151	99.93	1.10
33	4705632.3	04/02/2014	Chinstrap	guard	100	0	2014_2nd	2013/14	43,234	99.97	1.21
34	4705639.3	17/02/2014	Chinstrap	creche	100	0	2014_3rd	2013/14	44,626	99.98	1.44
35	4705449.3	17/02/2014	Chinstrap	creche	100	0	2014_3rd	2013/14	36,689	99.98	1.04
36	4715569.3	17/02/2014	Chinstrap	creche	89	11	2014_3rd	2013/14	119,839	99.96	1.46

37	4705455.3	29/12/2014	Adélie	guard	7	93	2015_1st	2014/15	33,674	99.95	1.21
38	4705638.3	29/12/2014	Adélie	guard	3	97	2015_1st	2014/15	55,682	99.95	1.14
39	4705557.3	29/12/2014	Adélie	guard	4	84	2015_1st	2014/15	41,426	99.94	0.92
40	4705497.3	09/01/2015	Adélie	creche	100	0	2015_2nd	2014/15	36,302	99.89	0.96
41	4705602.3	09/01/2015	Adélie	creche	100	0	2015_2nd	2014/15	56,057	99.96	1.76
42	4705489.3	09/01/2015	Adélie	creche	100	0	2015_2nd	2014/15	46,092	99.96	1.71
43	4705596.3	19/01/2015	Adélie	creche	100	0	2015_3rd	2014/15	90,599	99.96	1.36
44	4705550.3	19/01/2015	Adélie	creche	100	0	2015_3rd	2014/15	80,003	99.94	1.33
45	4705565.3	19/01/2015	Adélie	creche	100	0	2015_3rd	2014/15	13,375	99.97	1.51
46	4705466.3	22/01/2015	Chinstrap	guard	100	0	2015_1st	2014/15	22,071	99.95	1.10
47	4705547.3	22/01/2015	Chinstrap	guard	100	0	2015_1st	2014/15	72,261	99.94	1.13
48	4705482.3	22/01/2015	Chinstrap	guard	100	0	2015_1st	2014/15	51,970	99.94	0.81
49	4705517.3	05/02/2015	Chinstrap	guard	100	0	2015_2nd	2014/15	29,691	99.98	1.38
50	4705572.3	05/02/2015	Chinstrap	guard	100	0	2015_2nd	2014/15	30,169	99.93	1.46
51	4705544.3	05/02/2015	Chinstrap	guard	100	0	2015_2nd	2014/15	36,213	99.94	1.50
52	4705554.3	19/02/2015	Chinstrap	creche	100	0	2015_3rd	2014/15	34,470	99.95	1.27
53	4705513.3	19/02/2015	Chinstrap	creche	100	0	2015_3rd	2014/15	58,695	99.94	1.77
54	4705561.3	19/02/2015	Chinstrap	creche	100	0	2015_3rd	2014/15	47,113	99.94	0.97

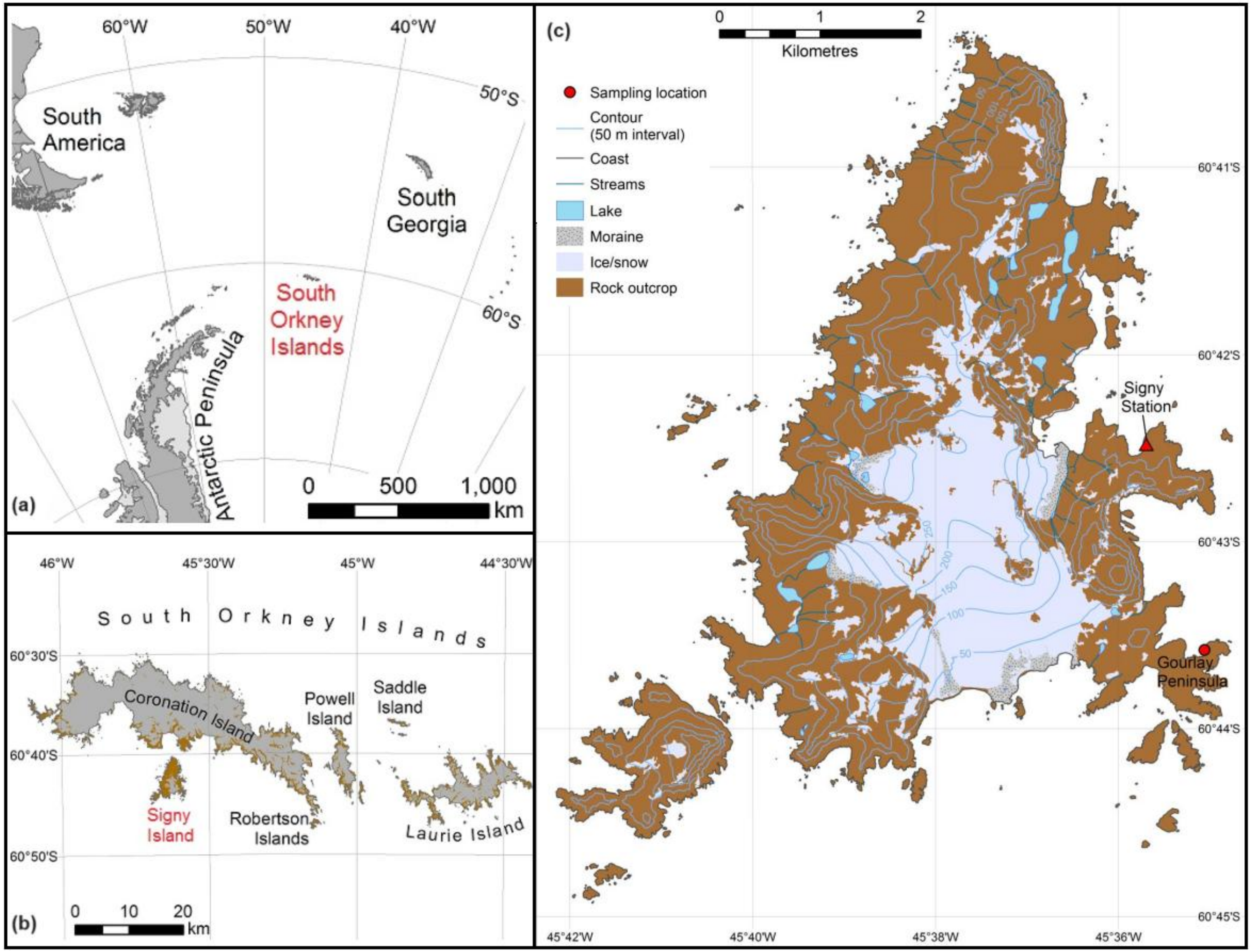

Fig. S1. The locations of (a) South Orkney Islands in the maritime Antarctic; (b) Signy Island within the South Orkney Island archipelago; and (c) Gourlay Peninsula on Signy Island. Map prepared by Laura Gerrish, Mapping and Geographic Information Centre, British Antarctic Survey.

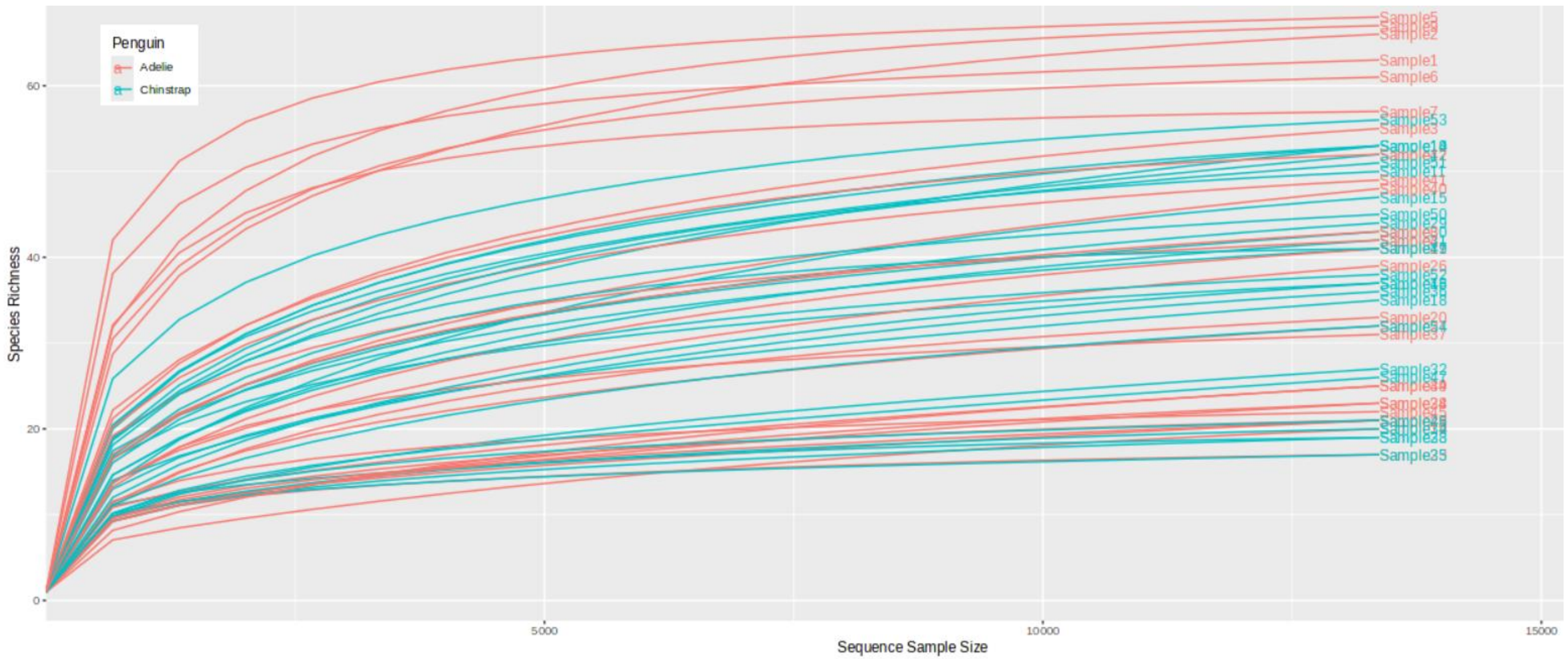

Fig. S2. Rarefaction analyses of all 54 samples that were successfully filtered and normalised to a minimum library size of 13,375 sequences.

Table S2. Statistical tests of Adélie and Chinstrap stomach bacterial alpha and beta diversity values between the comparison groups.

			Alpha diversity [a]	Beta diversity [b]
Inter-species		Season 2011/12 (n = 18 in total)	*p = 0.01	*F = 4.20, R2 = 0.21, p = 0.01
		Season 2013/14 (n = 18)	p = 0.11	F = 0.29, R2 = 0.02, p = 0.75
		Season 2014/15 (n = 18)	p = 0.89	F = 1.89, R2 = 0.11, p = 0.15
		Across seasons (n = 54)	p = 0.64	F = 2.54, R2 = 0.05, p = 0.07
Intra-season	Adélie penguins	Season 2011/12 (n = 9)	p = 0.56	F = 1.06, R2 = 0.26, p = 0.38
		Season 2013/14 (n = 9)	p = 0.11	F = 1.34, R2 = 0.31, p = 0.31
		Season 2014/15 (n = 9)	p = 0.19	F = 3.29, R2 = 0.52, p = 0.05
		Across seasons (n = 27)	p = 0.77	F = 1.81, R2 = 0.14, p = 0.12
	Chinstrap penguins	Season 2011/12 (n = 9)	p = 0.59	F = 2.86, R2 = 0.49, p = 0.07
		Season 2013/14 (n = 9)	p = 0.56	F = 3.41, R2 = 0.53, p = 0.12
		Season 2014/15 (n = 9)	p = 0.15	F = 1.29, R2 = 0.30, p = 0.32
		Across seasons (n = 27)	p = 0.24	F = 2.36, R2 = 0.16, p = 0.09
	Both *Pygoscelis* penguins (n = 54)		P = 0.22	*F = 3.65, R2 = 0.13, p < 0.01
Inter-season	Adélie penguins (n = 27)		*p < 0.01	*F = 3.48, R2 = 0.23, p = 0.01
	Chinstrap penguins (n = 27)		p = 0.24	F = 2.37, R2 = 0.17, p = 0.09
	Both *Pygoscelis* penguins (n = 54)		*p < 0.01	*F = 4.81, R2 = 0.16, p < 0.01
Diet	Adélie penguins (n = 27)		*p = 0.04	F = 1.43, R2 = 0.06, p = 0.25
Chick rearing stage	Adélie penguins	Season 2011/12 (n = 9)	p = 0.55	F = 1.34, R2 = 0.16, p = 0.27
		Season 2013/14 (n = 9)	-	-
		Season 2014/15 (n = 9)	p = 0.14	F = 2.82, R2 = 0.29, p = 0.12
		Across seasons (n = 27)	p = 0.88	F = 1.84, R2 = 0.07, p = 0.13
	Chinstrap penguins	Season 2011/12 (n = 9)	p = 0.72	*F = 3.98, R2 = 0.36, p = 0.03
		Season 2013/14 (n = 9)	p = 0.38	F = 2.12, R2 = 0.23, p = 0.18
		Season 2014/15 (n = 9)	p = 0.91	F = 2.27, R2 = 0.25, p = 0.13
		Across seasons (n = 27)	p = 0.63	F = 1.90, R2 = 0.07, p = 0.17
	Both *Pygoscelis* penguins (n = 54)		P = 0.59	*F = 3.36, R2 = 0.06, p = 0.02

[a] Mann-Whitney for inter-species, penguin diet and chick stage comparisons or Kruskal-Wallis for intra- and inter-seasonal comparisons

[b] PERMANOVA test

* Significant p < 0.05
